# Prolonged SARS-CoV-2 RNA detection by RT-PCR: variability in cycle threshold values over time

**DOI:** 10.1128/spectrum.01225-25

**Published:** 2025-09-08

**Authors:** Irene Muñoz-Gallego, Amparo Rubio, Miriam Maseda, Patricia Carnicero, Lola Folgueira

**Affiliations:** 1 Laboratoy of Virology, Microbiology Department, Hospital Universitario 12 de Octubre 16473, Madrid, Spain; 2 Biomedical Research Institute imas12, Hospital Universitario 12 de Octubre 16473, Madrid, Spain; 3 Department of Medicine, School of Medicine, Universidad Complutense 16734, Madrid, Spain; National Microbiology Laboratory, Winnipeg, Manitoba, Canada

**Keywords:** COVID-19, SARS-CoV-2, RT-PCR, RNA persistence, cycle threshold (Ct)

## Abstract

Millions of reverse transcription-polymerase chain reaction (RT-PCR) tests have been performed worldwide during the SARS-CoV-2 pandemic, using various protocols. This study evaluates the duration of SARS-CoV-2 RNA detectability by RT-PCR at body temperature and analyzes changes in cycle threshold (Ct) values over time. Positive nasopharyngeal swabs for SARS-CoV-2 RT-PCR (*n* = 120) with different Ct values were collected from Hospital Universitario 12 de Octubre (Madrid, Spain, 2020). Samples were processed using an open-access protocol for the Hologic Panther Fusion system, targeting the envelope gene. They were stratified into six Ct value groups (<20, 20.0–25.0, 25.1–30.0, 30.1–35.0, 35.1–40.0, and >40.0) and incubated at 37°C for 15, 30, 45, and 60 days, with RT-PCR repeated at each interval. Samples with Ct <20.0 remained positive throughout the study, whereas those with Ct >40.0 were negative by day 15. A significant proportion of samples with Ct ≤35.0 remained positive at day 60. Positivity during incubation decreased with higher Ct values (*P* < 0.001). Ct values increased over time but inconsistently by day 60. In conclusion, SARS-CoV-2 RNA can persist for months, leading to prolonged RT-PCR positivity. However, repeated RT-PCR testing introduces variability in qualitative results and Ct values, complicating clinical decisions. Patient monitoring should emphasize clinical assessment and viral viability assays rather than repeated RT-PCR testing.

**IMPORTANCE:**

This study investigates the prolonged persistence of SARS-CoV-2 detected by reverse transcription-PCR (RT-PCR) at human body temperature and the evolution of cycle threshold (Ct) values in positive respiratory samples. Our findings indicate that SARS-CoV-2 RNA can persist for months, and that repeated RT-PCR testing over time introduces variability, particularly in samples with high Ct values. This work highlights the necessity of complementing RT-PCR testing with methods assessing viral viability to avoid over-reliance on repeated testing in clinical decision-making. This study addresses a critical issue in COVID-19 diagnostics, with important implications for clinical management and infection control.

## OBSERVATION

SARS-CoV-2, the causative agent of coronavirus disease 2019 (COVID-19), emerged in late 2019 and rapidly spread, leading to a global pandemic (1). Reverse transcription-polymerase chain reaction (RT-PCR) became the diagnostic gold standard, with millions of tests performed worldwide. However, SARS-CoV-2 RNA can persist in the upper respiratory tract for weeks, complicating decisions regarding isolation discontinuation and return-to-work protocols (2).

Although multiple RT-PCR protocols have emerged focusing on the RT-PCR technique and its cycle threshold (Ct) value for monitoring COVID-19 patients, a positive result does not necessarily indicate the presence of viable virus. Nevertheless, negative RT-PCR results are sometimes required for specific situations, such as nursing home admissions.

This study aimed to evaluate the duration of SARS-CoV-2 RNA detectability at human body temperature and analyze Ct value variability over time.

A total of 120 SARS-CoV-2-positive nasopharyngeal swab samples with varying Ct values were collected from patients who attended the Emergency Department of Hospital Universitario 12 de Octubre (Madrid, Spain) between October and November 2020. Samples had been processed using an open-access protocol for the Hologic Panther Fusion system (San Diego, CA, USA), targeting the envelope gene (E) (limit of detection: 1 × 10^−2^ TCID_50_/mL) (3). Subsequently, the samples were frozen at −80°C until further analysis. They were selected based on their Ct values and grouped into six Ct ranges: <20.0 (*n* = 10), 20.0–25.0 (*n* = 25), 25.1–30.0 (*n* = 25), 30.1–35.0 (*n* = 25), 35.1–40.0 (*n* = 25), and ≥40.0 (*n* = 10). The study complied with ethical guidelines, and approval from a Research Ethics Committee was not required under Organic Law 3/2018.

For each sample, 0.5 mL of Universal Transport Medium (UTM) (Copan Diagnostics, Murrieta, CA, USA) was replaced with 0.5 mL of sample. RT-PCR was performed to assess the dilution effect, which resulted in 15 samples testing negative: 30.0–35.0 (*n* = 1), 35.0–40.0 (*n* = 7), and >40.0 (*n* = 7). The remaining 105 diluted samples were incubated at 37°C, the standardized core body temperature, and retested at 15, 30, 45, and 60 days. If a sample turned negative, RT-PCR was repeated after 15 days; if it remained negative, no further tests were performed.

The percentage of positive samples and their median Ct values with interquartile range (IQR) were analyzed over time. Continuous variables were compared using the Student’s *t*-test or the Mann–Whitney *U*-test, while categorical data were analyzed with chi-square or Fisher’s exact test. Statistical significance was defined at *P* ≤ 0.05. All statistical analyses were performed using SPSS Version 20.0 (IBM Corp., Armonk, NY, USA).

The percentage of positive samples and corresponding Ct values is summarized in Table 1. All diluted samples with Ct <20.0 remained positive throughout the study, while those with Ct >40.0 were negative by day 15. Additionally, all samples with Ct ≤25.0 were positive by day 15, whereas those with Ct ≥35.1 turned negative by day 60. A significant proportion of diluted samples with Ct ≤35.0 remained positive at day 60: 100% (2/2) of those with Ct <20.0, 43.8% (7/16) with Ct 20.0–25.0, 18.5% (5/27) with Ct 25.1–30.0, and 13.3% (4/30) with Ct 30.1–35.0. As shown in Table 1, positivity rates decreased significantly with higher Ct values during incubation (*P* < 0.001).

**TABLE 1:** Percentage of SARS-CoV-2 RT-PCR positivity and corresponding Ct values in diluted samples across different days of incubation at 37°C

Ct of positive diluted samples (*N* = 105)	Diluted		Day 15		Day 30		Day 45		Day 60	
	Positivity (%)	Ct (median [IQR])^*b*^	Positivity (%)	Ct (median [IQR])	Positivity (%)	Ct (median [IQR])	Positivity (%)	Ct (median [IQR])	Positivity (%)	Ct (median [IQR])
<20.0 (*N* = 2)	2 (100.0)	17.8 (17.8–17.8)	2 (100.0)	21.9 (19.4–21.9)	2 (100.0)	23.7 (20.1-23.7)	2 (100.0)	30.8 (21.0–30.8)	2 (100.0)	28.3 (20.6–28.3)
20.0–25.0 (*N* = 16)	16 (100.0)	23.0 (21.8–23.9)	16 (100.0)	28.6 (27.1–30.4)	16 (96.0)	30.9 (30.2–32.3)	15 (93.8)	34.1 (30.9–39.0)	7 (43.8)	33.7 (33.1–37.2)
25.1–30.0 (*N* = 27)	27 (100.0)	27.1 (26.1–28.7)	27 (100.0)	33.0 (31.1–34.7)	26 (96.3)	35.9 (34.1–37.1)	11 (40.7)	36.9 (34.1–37.7)	5 (18.5)	36.8 (33.4–41.3)
30.1–35.0 (*N* = 30)	30 (100.0)	32.6 (31.1–33.5)	26 (86.7)	38.2 (36.0–39.5)	18 (60.0)	38.5 (38.0–40.7)	8 (26.7)	38.9 (37.3–40.7)	4 (13.3)	38.8 (38.5–39.7)
35.1–40.0 (*N* = 22)	22 (100.0)	39.0 (36.7–39.4)	5 (22.7)	40.5 (40.0–41.4)	3 (13.6)	41.1 (40.0–41.1)	4 (18.2)	41.2 (39.4–42.9)	0 (0.0)	–^*a*^
>40.0 (*N* = 8)	8 (100.0)	40.6 (40.2–41.1)	0 (0.0)	–	0 (0.0)	–	0 (0.0)	–	0 (0.0)	–

^*a*^ –: Ct value is not available because of negative RT-PCR result.

^*b*^ IQR: interquartile range.

Median Ct values of positive samples increased over time, but were inconsistent at day 60 (Table 1). Ct values were significantly associated with initial Ct values (*P* < 0.001 at days 15 and 30, *P* = 0.020 at day 45) except at day 60 (*P* = 0.063). Significant differences in Ct values were also observed over time. In the Ct 20.0–25.0 group, differences were found between day 15 and days 30, 45, and 60 (*P* < 0.001), as well as between day 30 and days 45 and 60 (*P* = 0.006 and *P* = 0.001, respectively). In the Ct 25.1–30.0 group, significant differences were observed between day 15 and days 30 and 45 (*P* < 0.001 and *P* = 0.003, respectively).

Of note, nine samples (8.6%) tested positive after initially turning negative, mostly in those with Ct >35.0 (5/9 samples [55.6%]). Additionally, Ct values decreased over time in 11 out of 105 samples (10.5%), particularly in those with Ct 30.0–35.0 (6/11 samples, 54.5%). Decreases were more pronounced in samples with lower baseline Ct values: 1.00 (0.40–3.35) versus 0.65 (0.50–0.83) for Ct ranges <30.0 and 30.1–35.0, respectively.

Our finding confirms that SARS-CoV-2 RNA can persist for months in samples with low Ct values (4). Although RNA detectability typically declines within 2 weeks, prolonged shedding has been reported, particularly in immunocompromised patients (5, 6). RT-PCR remains a highly sensitive method, capable of detecting both viable and non-viable viral RNA.

We observed a progressive increase in Ct values over time, consistent with previous studies on RT-PCR variability (7, 8). However, Ct cutoffs should be interpreted cautiously, as non-standardized values are insufficient for determining infectiousness.

The Infectious Diseases Society of America does not endorse the use of Ct values alone for clinical decision-making (9). Notably, some samples initially tested negative but later turned positive, particularly those near the assay detection limit. Late Ct values are defined as those near the upper limit of detection for a specific test and are associated with lower viral loads in the sample. Typically, these values are within three to five cycles of the Ct cutoff threshold and often are in the high 30s to mid-40s range (5). These fluctuations likely reflect RNA persistence rather than reinfection. Additionally, in some cases, Ct values decreased over time, complicating result interpretation.

Given these findings, patient monitoring should prioritize clinical assessment and methods that evaluate viral viability over repeated RT-PCR testing. Viral culture remains the gold standard for assessing infectivity, but it is not routinely performed in clinical laboratories.

This study has certain limitations, including the use of thawed samples, which might have influenced results. First of all, the open-access protocol for the Hologic Panther Fusion system does not include controls; therefore, strict precautions were implemented to prevent cross-contamination. The lack of viral culture prevents direct assessment of infectivity. Additionally, the RT-PCR protocol used was not CE-marked. Different SARS-CoV-2 variants of concern were not included, as they emerged later in the pandemic. Despite these limitations, our findings provide valuable insights into RT-PCR dynamics and the persistence of SARS-CoV-2 RNA.

In conclusion, this *in vitro* study confirms that SARS-CoV-2 RNA can persist for months, leading to prolonged RT-PCR positivity. However, repeated RT-PCR testing over time may introduce variability in both qualitative results and Ct values, particularly at higher Ct ranges near the detection limit. Given these fluctuations, patient monitoring should emphasize clinical assessment and viral viability rather than relying solely on sequential RT-PCR testing. Future research should focus on integrating alternative methods for assessing infectivity and disease severity in clinical decision-making.

## Data Availability

The data presented in this study are available in the article.
